# Impairment and Differential Expression of PR3 and MPO on Peripheral Myelomonocytic Cells with Endothelial Properties in Granulomatosis with Polyangiitis 

**DOI:** 10.1155/2012/715049

**Published:** 2012-06-26

**Authors:** Susann Patschan, Daniel Patschan, Elvira Henze, Sabine Blaschke, Johannes T. Wessels, Gerhard Anton Müller

**Affiliations:** ^1^Department of Nephrology and Rheumatology, University Medical Center Göttingen, 37075 Göttingen, Germany; ^2^Core Facility “Molecular & Optical Live Cell Imaging (MOLCI)”, University Medical Center Göttingen, 37075 Göttingen, Germany

## Abstract

*Background*. Granulomatosis with polyangiitis (GPA) and microscopic polyangiitis (MPA) are autoimmune-mediated diseases characterized by vasculitic inflammation of respiratory tract and kidneys. Clinical observations indicated a strong association between disease activity and serum levels of certain types of autoantibodies (antineutrophil cytoplasm antibodies with cytoplasmic [cANCA in GPA] or perinuclear [pAN CA in MPA] immunofluorescence). Pathologically, both diseases are characterized by severe microvascular endothelial cell damage. Early endothelial outgrowth cells (eEOCs) have been shown to be critically involved in neovascularization under both physiological and pathological condition. *Objectives*. The principal aims of our study were (i) to analyze the regenerative activity of the eEOC system and (ii) to determine mPR3 and MPO expression in myelo monocytic cells with endothelial characteristics in GPA and MPA patients. *Methods*. In 27 GPA and 10 MPA patients, regenerative activity blood-derived eEOCs were analyzed using a culture-forming assay. Flk-1^+^, CD133^+^/Flk-1^+^, mPR3^+^, and Flk-1^+^/mPR3^+^ myelomonocytic cells were quantified by FACS analysis. Serum levels of Angiopoietin-1 and TNF-*α* were measured by ELISA. *Results*. We found reduced eEOC regeneration, accompanied by lower serum levels of Angiopoietin-1 in GPA patients as compared to healthy controls. In addition, the total numbers of Flk-1^+^ myelomonocytic cells in the peripheral circulation were decreased. Membrane PR3 expression was significantly higher in total as well as in Flk-1^+^ myelomonocytic cells. Expression of MPO was not different between the groups. *Conclusions*. These data suggest impairment of the eEOC system and a possible role for PR3 in this process in patients suffering from GPA.

## 1. Introduction

Granulomatosis with polyangiitis (GPA) and microscopic polyangiitis (MPA) are autoimmune diseases characterized by systemic necrotizing vasculitis mainly affecting the respiratory tract and the kidneys [1]. Histopathological analysis reveals severe structural alterations of microvascular endothelial cells, leading to impaired microcirculation in skin, joints, respiratory tract, and kidneys, respectively. Endothelial damage has been suggested to result from interactions between primed neutrophils and ANCA with the endothelium followed by endothelial detachment from the basement membrane [2]. A number of different studies showed increased levels of both, circulating mature endothelial cells and endothelial microparticles in GPA and MPA [3, 4]. Thus, patients with GPA and MPA suffer from ongoing vascular damage which can be controlled by immunosuppressive treatment. New mechanisms of microvascular repair have been elucidated in recent years. So-called endothelial progenitor cells (EPCs), heterogenous in nature and initially been identified by Asahara et al. in 1997 [5], have been documented to promote endothelial repair under both physiological and pathological conditions [6–10]. According to the current concept on EPC biology, the cells are represented by two major populations, early and late endothelial outgrowth cells [11, 12]. Early endothelial outgrowth cells (eEOCs) have also been defined as myelomonytic (hematopoietic) cells with proangiogenic properties [11]. Impaired eEOCs regeneration and activity have been shown in different types of noninflammatory and inflammatory vascular diseases: patients with ANCA-associated vasculitis (AAV) showed increased numbers of circulating CD34^+^ hematopoietic progenitor cells (HPCs) and endothelial progenitor cells (EPCs) after the institution of immunosuppressive therapy and disease remission [13]. A recent study also showed a significant and persistent deficiency of circulating EPCs (eEOCs) in patients with AAV. The authors concluded that low EPC numbers could potentially reflect an impaired mechanism of vascular repair and may contribute to repeated relapses of the disease [14]. In GPA and MPA, antibodies directed againts PR3 and MPO have become important tools in diagnosing the diseases and in monitoring disease activities under treatment [15]. Elevated expression levels of membrane-bound PR3 (mPR3) have been observed in GPA and some other chronic inflammatory diseases, suggesting a pathogenic role of mPR3 by allowing interaction with PR3-ANCA [16]. ANCA binding to target antigens with subsequent activation of neutrophils resulting in premature degranulation and endothelial cell damage (ANCA-cytokine sequence theory) has been (and still is) controversially discussed as key event in the pathogenesis of GPA [17]. Recently, myelomonocytic cells have been shown to increase PR3 gene transcription in small vessel vasculitis as well [4]. In addition, high anti-PR3 concentration in patients with acute vasculitis has been shown to correlate with an activated adhesion molecule phenotype in circulating monocytes [18].

Therefore, the principal goals of our study were to analyze the regenerative activity of the eEOC system in GPA and MPA patients and to determine mPR3 and MPO expression in myelomonocytic cells with endothelial properties.

## 2. Patients and Methods

### 2.1. Patients and Blood Samples

Blood samples were obtained from 25 GPA and from 10 MPA patients. Patients' baseline characteristics are summarized in Table 1. All patients were recruited in the Department of Nephrology and Rheumatology at the University Medical Center Göttingen over a period of 15 months. The study protocol was approved after review by the local ethics committee. The investigation conformed to the principles outlined in the Declaration of Helsinki and written informed consent was obtained from each subject. All GPA patients met the criteria previously established by the American College of Rheumatology [19] and were tested positive for cANCA at least once in their history. Disease activity was scored by using the validated Birmingham Vasculitis Score (BVAS) [20]. A BVAS of 0 with a prednisolone dose below or equal to 7.5 mg/d defined remission while an active disease was defined by a BVAS ≥ 1. Patients with a BVAS of ≥8 were defined as highly active, a BVAS of <8 defined low activity. MPA was diagnosed by clinical characteristics, detection of pANCA, and presence of active glomerulonephritis in renal biopsy. Disease activity in MPA patients was evaluated by assessment of clinical symptoms (hemoptysis, purpura), urine analysis (red blood cell casts, acanthocytes), and measurement of CRP and/or ESR. Patients were initially treated by pulse cyclophosphamide therapy (10–15 mg/kg adapted to the level of renal function) with or without plasmapheresis and oral or intravenous corticosteroids. Patients in remission were treated by either azathioprine, mycophenolate mofetil, methotrexate, and/or low-dose prednisone (dosage: ≤7.5 mg). One patient with GPA received leflunomide for maintenance therapy. Healthy, age- and gender-matched individuals served as controls. In addition, a total number of 19 patients with systemic lupus erythematosus (SLE) were included into the study in order to perform control measurements. SLE was diagnosed according to the criteria established by the American College of Rheumatology [21]. Activity in SLE was assessed by the SLEDAI (systemic lupus erythematosus disease activity index [22]). For the studies, each patient (and the respective controls) provided four blood samples (7.5 mL each), from which two (2 × 7.5 mL) were employed for endothelial and myelomonocytic cell studies, and two (2 × 7.5 mL) were used for performing routine laboratory (see *Biochemical and hematological tests*) as well as immunological studies. For quantifiying renal function, serumcreatinin was measured and urine was collected for proteinuria [23].

### 2.2. Flow Cytometry

For performing flow cytometry, mononuclear cells (MNCs) were isolated by density gradient centrifugation using Histopaque-1077 solution (Sigma Diagnostics, St. Louis, MO) from *≈*7,5 mL of heparinized peripheral blood. Cells were primarily incubated for 1 hour on ice with one or more of the following antibodies: rabbit anti-CD133 (ab16518—Abcam, Cambridge, UK), mouse anti-human VEGFR2 (FAB 357F—R&D Systems, Minneapolis, MN, USA), mouse anti-c-Kit (3310, alexa flour 488—Cell Signaling, Boston, MA, USA), mouse anti-MPO (ab11730—Abcam, Cambridge, UK), mouse anti-PR3 (sc-52716—Santa Cruz, CA, USA), followed by secondary incubation with PE-conjugated goat-anti-rabbit Fab (CD133, 111-116-144—Jackson Immunoresearch, Baltimore, PA, USA), PE-conjugated goat anti-mouse (MPO, 11730-500, Abcam, Cambridge, UK), PE-conjugated goat anti-mouse (PR3, 115-116-146—Jackson Immunoresearch, Baltimore, PA, USA) for 30 minutes on ice, respectively. After incubation cells were washed with PBS-BSA 1% (w/v). Data were acquired using a FACScalibur cytometer (Becton Dickinson, Heidelberg, Germany) equipped with a 488 nm argon laser and a 635 nm red diode laser and analyzed using CellQuest software (Becton Dickinson, San Jose, CA, USA). The setup of FACScalibur was performed according to the manufacturer's instructions using unstained and single-antibody stained cells. Specificity of staining was controlled by incubation with isotype-matched immunoglobulins. To quantify total peripheral cells of the endothelial lineage, the numbers of Flk-1 positive cells, to quantify EPCs, the numbers of CD133/Flk-1 double-positive cells within the myelomonocytic cell population were counted [24, 25].

### 2.3. Analysis of EPC Proliferation (Colony-Forming Units (CFU) Assay)

The assay was performed by using the EndoCult Liquid Medium Kit (StemCell Technologies, Vancouver, BC, Canada) per the manufacturer's protocol. MNCs were resuspended in complete EndoCult medium and seeded at 5 × 10^6^ cells/well on fibronectin-coated tissue culture plates (BD Biosciences, Rockville, MD, USA). After 48 hours, wells were washed with media and nonadherent cells were collected. Nonadherent cells were plated in their existing media at 10^6^ cells/well in 24 well fibronectin-coated tissue culture plates for three days. Only colonies with at least 20 cells, containing rounded cells in the middle and elongated cells at the periphery, were considered as CFU-EC colonies. The number of colonies appearing after this period was counted [12]. At least two members of the laboratory staff evaluated the numbers of CFU-ECs. They were blinded for the diagnosis and status of the investigated patients/controls.

### 2.4. Immunofluorescence

In all patients, the phenotype of cells within the colonies was determined more in detail. For this purpose, cells were characterized by the uptake of DiI-labeled acetylated low density lipoprotein (acLDL) (Invitrogen, Carlsbad, CA, USA) and binding of FITC-labeled UE lectin (Sigma Diagnostics, St. Louis, MO). Cells were first incubated with 10 *μ*g/mL DiI-ac-LDL at 37°C for 1 h and later fixed with 2% formaldehyde for 10 min, followed by incubation with UE lectin at 37°C for 1 h. The number of Dil-acLDL^+^/UE lectin^+^ cells was counted by laser scanning microscopy using an inverted fluorescence microscope IX-71 (Olympus Deutschland GmbH, Hamburg, Germany) equipped with the appropriate excitation and emission filters (AHF Analysentechnik, Tuebingen, Germany). A second sample of 10^6^ cells from each patient was cultured on fibronectin-coated circular glass bottom slides and stained for Dil-acLDL uptake, UE lectin binding, and PR3 expression (for primary antibody see *Flow cytometry*, secondary antibodies: donkey anti-mouse IgG (NL-637, R&D Systems, Wiesbaden, Germany) and goat anti-mouse (Alexa Fluor 647—A21240, Invitrogen, Germany)). An average of 100 Dil-acLDL^+^/UE lectin^+^ cells per patient/control was analyzed for mPR3 expression. In order to analyze mPR3 expression per individual cell, single cell laser scanning microscopy was performed. Cells were examined using the inverted fluorescence microscope IX-71 (see above). Images of respective fluorescence channels were recorded as single high-resolution 16 bit b/w images using an F-View II ext. Camera (Olympus Deutschland GmbH, Hamburg, Germany). The images from every fluorescence channel were automatically merged using the MFIP-module of the CELL-F software. Representative images of Dil-acLDL^+^/UE lectin^+^/PR3^+^ cells cells were taken using the IX-71 fluorescence microscope (Olympus Deutschland GmbH, Hamburg, Germany).

### 2.5. Enzyme-Linked Immunosorbent Assay (ELISA)

Commercial ELISA tests were purchased for the assessment of IL-6 (IMMULITE, Siemens, Germany), TNF-*α*  (Siemens, Germany), VEGF and SDF-1 (USCN, Wuhan, China), and Angiopoietin-1 (Alpco, Salem, NH, USA) serum levels. ELISA tests were performed according to the manufacturer's protocol.

### 2.6. Western Blot Analysis

Cultured EPCs from healthy controls and GPA patients (*Analysis of EPC proliferation (colony-forming units (CFU) assay)*) were homogenized in Lysis buffer (1% (v/v) KPO_4_/EDTA, 5 mM EGTA, 10 mM MgCl_2_, 50 mM *β*-glycerolphosphate, 1 mM Na_3_VaO_4_, 0.5% (v/v) NP_4_O, 0.1% (v/v) Brij-35, 0.1% (v/v) PMSF, 0.01% (v/v) Leupeptin, 0.01% (v/v) Pepstatin A), followed by 15 minutes of incubation on ice. After centrifugation for 15 minutes at 2.000 rpm, pellets were resuspended in 100 *μ*L of PBS and protein concentration was measured according to a previously published protocol [26]. Sample volumens (100 *μ*L) were mixed with 400 *μ*L of methanol, 100 *μ*L of chloroform, and 300 *μ*L of destilled water. After centrifugation for 1 minute at 14.000 rpm proteins were collected and resuspended in 400 *μ*L of methanol. Centrifugation and resuspension in methanol were repeated once, after the third centrifugation the pellets were finally resuspended in SDS sample buffer. Equal amounts of protein (measured by extinction analysis, later confirmed by F-actin immunoblotting) were electrophoretically separated in 12.5% Trisglyceride gels (Invitrogen, Carlsbad, CA, USA) and transferred to immunoblot membranes (PVDF membranes—162-0177, BIO-RAD, Hercules, CA, USA). After blocking with TBS-T-containing nonfat dry milk (5% w/v), membranes were incubated with the primary antibody mouse anti-PR3 (sc-52716—Santa Cruz, CA, USA) overnight at 4°C. After washing with TBS-T, membranes were incubated with a horse-reddish peroxidase-conjugated secondary antibody (polyclonal rabbit-anti-mouse—P0161, 1 : 2000, DAKO, Carpenteria, CA, USA) for 60 minutes at room temperature. Membranes were washed and protein was detected using a chemiluminescence system (ECL Plus Western Blotting Detection System—RPN2132, GE Healthcare, Amersham, Piscataway, NJ, USA).

### 2.7. Biochemical and Hematological Tests

Biochemical and hematological tests were performed in the Central Laboratories of the University Hospital Göttingen, according to the institutional guidelines.

### 2.8. Statistical Analysis

All values are expressed as mean ± SEM. The means of two populations were compared by Mann-Whitney *U* test. Correlation analysis was performed by Spearman's rank correlation test. Differences between two groups were considered significant at *P* < 0.05, positive correlation was considered at *r* between 0.5–0.8.

## 3. Results

### 3.1. Circulating Myelomonocytic Cells with Endothelial Properties and Circulating eEOCs

**Figure 1:**
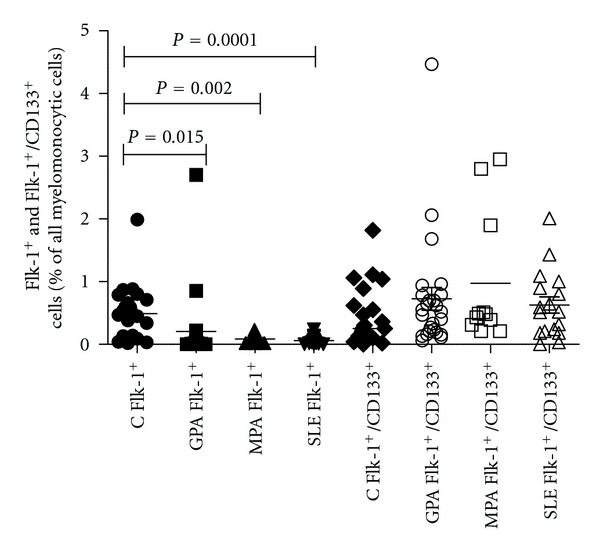
Flk-1^+^ and Flk-1^+^/CD133^+^ myelomonocytic cells (eEOCs) in patients with GPA, MPA, and SLE as compared to healthy controls. The percentages of circulating Flk-1^+^ cells (defined as all cells displaying endothelial characteristics) were lower in all patient groups (GPA, MPA, SLE) as compared to healthy controls. In contrast, total peripheral circulating eEOCs (Flk-1^+^/CD133^+^ cells) were not different between the four groups (bars show median).

The percentages of circulating myelomonocytic cells displaying endothelial characteristics were significantly lower in both groups of patients (GPA and MPA) as compared to controls (GPA: 0.19 ± 0.08% versus 0.52 ± 0.09%, *P* = 0.015, MPA: 0.08 ± 0.082% versus 0.52 ± 0.09%, *P* = 0.002 [% of total MNC]). In contrast, there were no differences in the numbers of circulating early endothelial outgrowth cells (eEOCs) between the three groups (Figure 1). All myelomonocytic cells expressing CD133 and Flk-1 were defined as circulating eEOCs [27].

### 3.2. Proliferative Activity of Circulating eEOCs (Number of CFU-ECs)

**Figure 2:**
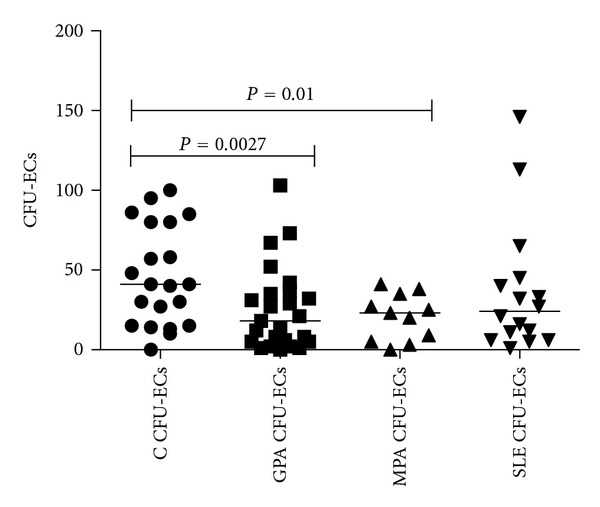
Proliferative activity of peripheral circulating eEOCs in GPA, MPA, and SLE. Patients with either GPA or MPA showed significantly less eEOC proliferation (defined as smaller numbers of colony unit endothelial cells [CFU-ECs]) than controls (bars show median). SLE patients were not different from controls. In GPA the numbers of colonies did not correlate with clinical activity of the disease as represented by BVAS (not shown).

Previous own studies [27] and analyses performed by others [28] had shown that circumstances characterized by vascular damage are associated with impaired endothelial progenitor cell regeneration. Therefore, in order to assess the regenerative potential of the eEOC system, a colony-forming unit (CFU) assay was performed. It clearly showed lower numbers of colonies in both groups, GPA and MPA patients, as compared to healthy controls (24.3 ± 5.1 and 22.5 ± 4.3 versus 45.9 ± 6.8, *P* = 0.0027 and *P* = 0.01) (Figure 2). In GPA, there was no linear correlation between the numbers of colonies formed in culture and clinical activity of the disease as reflected by the BVAS. In addition, colony forming did also not correlate with either CRP, IL-6, or BVAS (data not shown). Finally, Spearman's rank correlation test was performed in order to analyze whether suppressed colony forming by eEOCs correlates with renal function. Our analysis did not show a linear correlation between the two parameters.

### 3.3. Serum Levels of Angiopoietin-1

Angiopoietin-1 (Ang-1) is a ligand for endothelium-specific receptor tyrosine kinase Tie-2. In adult vasculature, Ang-1/Tie-2 signaling is thought to regulate both maintenance of vascular quiescence and promotion of angiogenesis [29]. Endothelial progenitor cell-mediated therapeutic neovascularization has been shown to be augmented by combined VEGF(165)/Angiopoietin-1 activation [30]. Since our results suggested significant impairment of myelomonocytic cells with endothelial properties and of eEOCs, Angiopoietin-1 serum levels were measured and compared to those of healthy controls. Serum Angiopoietin-1 levels were in fact lower in patients with GPA as compared to controls (1542 ± 315 pg/mL versus 689 ± 224 pg/mL, *P* = 0.034). There were no differences for serum vascular endothelial growth factor (VEGF) or serum stromal cell-derived factor-1 (SDF-1).

### 3.4. Expression of PR3 and MPO on Peripheral Cells of the Endothelial Lineage

**Figure 3:**
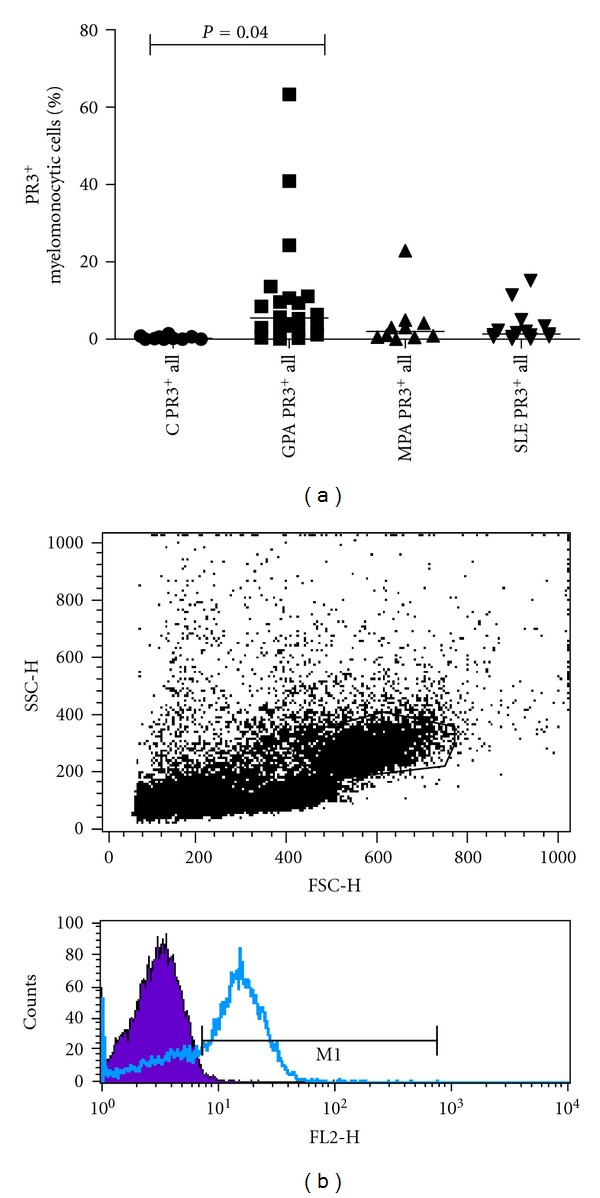
Percentages of all PR3^+^ myelomonocytic cells. GPA patients displayed higher percentages of cells expressing Proteinase 3. MPA and SLE patients were not different from controls ((a), bars show median). Images in (b) show representative FACS plots of a patient with GPA. The upper image in (b) displays the gating strategy for analyzing myelomonocytic cells, the lower image indicates increased percentages of mPR3 positive cells.

**Figure 4:**
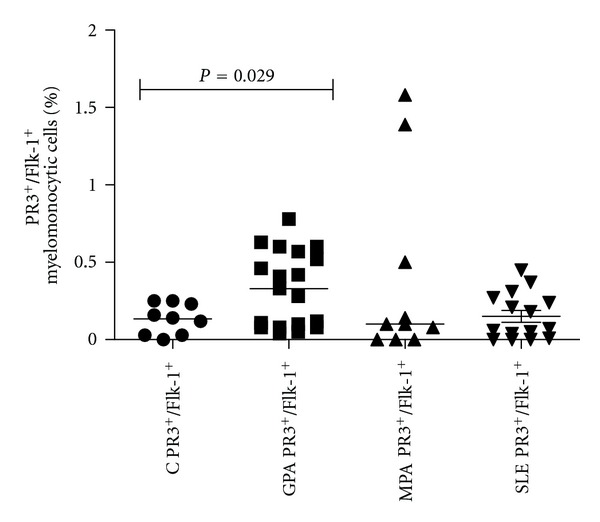
Percentages of PR3^+^/Flk-1^+^ cells. In order to evaluate PR3 expression on cells belonging to the endothelial lineage, peripheral myelomonocytic cells were stained for both, PR3 and Flk-1. As for the total myelomonocytic cell population, GPA patients showed higher percentages of PR3^+^/Flk-1^+^ cells than controls. Such differences were not detected between MPA or SLE and controls (bars show median).

**Figure 5:**
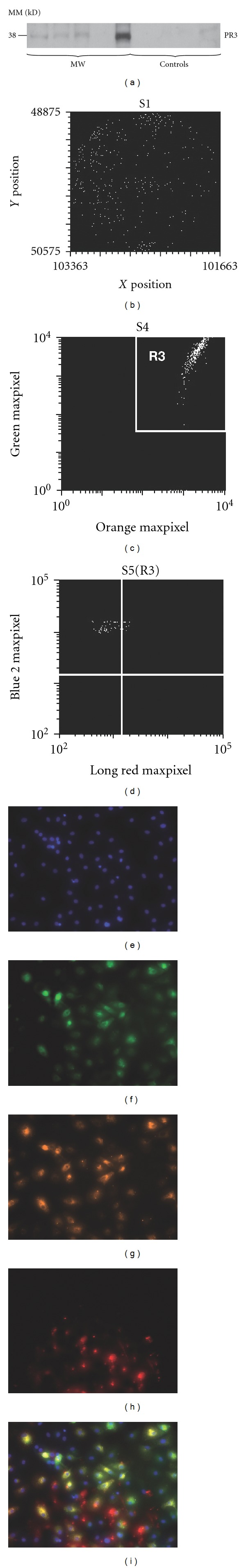
(a) Immunoblot analysis of eEOC plasma membrane preparations for mPR3 expression. All but one out of five GPA patients displayed significant mPR3 expression. The one individual with intense mPR3 signal was a patient that refused therapy from the beginning of the disease. (b)–(d) Laser scanning analysis of cultures eEOCs. The cells were detected by UE lectin binding (green immunofluorescence in (c)) and Dil-acLDL uptake lectin binding (red immunofluorescence in (c)). All double-positive cells were analyzed for mPR3 expression (long red in (d)). Only a minority of the cells was mPR3 positive. (e)–(i) Representative images of PR3 expression in cultured peripheral eEOCs. Cells were stained for UE lectin ((f)—green), Dil-acLDL uptake ((g)—orange), and PR3 ((h)—red). Image e merges images (e)–(h) ((e): nuclear staining with DAPI solution, magnification in (e)–(i) × 100).

Since our results showed (i) lower percentages of Flk-1^+^ myelomonocytic cells, (ii) depressed eEOC regeneration, and (iii) suppression of endothelial cell stimulating Angiopoietin-1, we sought to determine mPR3 and MPO expression in the total as well as in the Flk-1^+^ myelomonocytic cell population by flow cytometry. In both cell populations, percentages of mPR3^+^ cells were higher in GPA than in healthy controls (10 ± 3.1% versus 0.38 ± 0.14%, *P* = 0.04 and 0.33 ± 0.05% versus 0.13 ± 0.03%, *P* = 0.029). This was not the case in MPA (Figures 3 and 4). In contrast, MPO expression did not differ between the three groups. In order to further confirm the data on mPR3 expression, cultured eEOCs from healthy controls and GPA patients were analyzed for membrane-bound mPR3 by Western Blot analysis. All but one out of five analyzed GPA patients displayed significant mPR3 expression, whereas healthy controls were mPR3 negative (Figure 5). The same figure also shows representative immunofluorescence images and laser scanning plots of peripheral blood-derived endothelial progenitor cells from patients with GPA. As suggested by the flow cytometric data, only a minority of eEOCs were shown to express mPR3 (Figure 5). Finally, patients systemic lupus erythematosus were analyzed as well. In these patients, mPR3 expression was not higher than in healthy controls.

### 3.5. Serum Levels of Tumor Necrosis Factor-*α*  (TNF-*α*)

An increased membrane expression of PR3 during neutrophil adhesion has been shown to occur after TNF-*α*  stimulation [31]. We, therefore, sought to determine serum TNF-*α*  levels in GPA patients as compared to controls. GPA patients showed in fact significantly higher mean TNF-*α*  levels (13 ± 1 versus 8.9 ± 0.5 pg/mL, *P* = 0.04). No linear correlations were found between serum TNF-*α*  and CFU-ECs, mPR3 and mPR3/Flk-1 expression on myelomonocytic cells, as well as between TNF-*α*  and CD133^+^/Flk-1^+^ cells.

## 4. Discussion

Aims of the current study were to analyze the regenerative activity of the eEOC system in GPA and MPA patients and to determine mPR3 and MPO expression in myelomonocytic cells with endothelial properties. We found lower numbers of circulating Flk-1^+^ myelomonocytic cells without any differences in the percentages of peripheral circulating early endothelial outgrowth cells (CD133^+^/Flk-1^+^ myelomonocytic cells). In addition, GPA and MPA patients displayed lower proliferative activity of blood-derived eEOCs (lower numbers of colonies formed in culture—CFU assay). This was in line with observations made by Závada and colleagues [14] who showed significantly decreased CFU-ECs (colony-forming unit endothelial cells) in patients with ANCA-associated vasculitis (AAV). The lower percentages of Flk-1^+^ myelomonocytic cells despite physiological levels of CD133^+^/Flk-1^+^ myelomonocytic cells might result from a general defect of endothelial differentiation which possibly not only involves *de novo* generation of eEOCs in the bone marrow but also further differentiation of circulating CD133^+^/Flk-1^+^ cells into Flk-1^+^ myelomonocytic cells. There was no correlation between decreased formation of eEOC colonies and renal function as it had been shown in earlier studies [28]. Such conflicting data may be due to patient's characteristics since most patients included into our study did not suffer from severe renal insufficiency. Since total CD133^+^ cells were higher in GPA patients than in controls, the normal percentages of CD133^+^/Flk-1^+^ cells in GPA could also result from a *compensatory* increase in bone marrow/blood CD133^+^ cell proliferation. Prominin 1 (CD133) is known to be expressed in hematopoietic stem/progenitor cells (HSCs/HPCs) [32–34]. Although we did not find higher percentages of CD133^+^/c-Kit^+^ cells (which should foremost represent *immature *hematopoietic progenitor cells) in GPA as compared to controls (data not shown), increases in CD133^+^ cells could reflect mobilization of more differentiated HPCs in AAV. Increased mobilization of CD34^+^ hematopoietic progenitor cells has in fact been shown to occur in AAV after induction of remission [13].

The impaired regeneration and differentiation of the eEOC system were accompanied by lower blood levels of endothelial cell stimulating Angiopoietin-1. To our knowledge, this is the first study showing decreased serum Angiopoietin-1 in patients with GPA, potentially reflecting impaired neovascularization. The mechanisms responsible for suppression of the endothelial system in GPA still remain a matter of debate. Circulating Angiopoietin-2 (Ang-2) concentrations have been shown to be increased in patients with active SLE, while Angiopoietin-1 (Ang-1) was decreased [35]. Both mediators, Ang-1 and -2, can act agonistic as well as antagonistic in the process of vascular repair. Ang-1 stimulates endothelial proliferation, differentiation, and migration [36], whereas Ang-2 has been documented to disrupt vasoprotective Ang1/Tie2 signalling in some situations [35].

Another possible promoter of endothelial dysfunction in GPA is TNF-*α*. Tumor necrosis factor-*α*  mediates endothelial cell apoptosis by various mechanisms including caspase-3 and -8 activation, and stimulation of PKCbeta(2) [37–40]. GPA patients displayed higher mean serum TNF-*α*  than controls, which indicates its potential involvement in endothelial alteration. However, TNF-*α*  did not correlate with either the numbers of endothelial colonies formed in culture or with the percentages of CD133^+^/Flk-1^+^ cells in the blood. Therefore, the significance of increased serum TNF-*α*  levels in GPA remains speculative. Nevertheless, TNF-*α*  blocking agents such as infliximab have been used in the treatment of refractory cases of GPA [41] and it has been shown that pretreatment of cells with TNF-*α*  induces PR3 membrane expression and secretion [31].

In general, influences of the different immunomodulatory agents that were used for treating GPA patients on endothelial proliferation/regeneration cannot be exclusively ruled out. Nevertheless, cyclophosphamide has been reported to act as EPC mobilizing agent [42], and steroids have been documented to significantly increase circulating EPCs [43].

Our particular interest in myelomonocytic cells resulted from the fact that these cells have been shown to increase PR3 gene transcription in small vessel vasculitis [4]. In addition, intracellular trafficking of PR3 in myelomonocytic cells has been analyzed in earlier studies [44]. On the other hand, both cell types, mature myelomonocytic and early endothelial outgrowth cells, are most likely derived from pluripotent hematopoietic stem cells in the bone marrow and they both have been shown to act on mature endothelial cells in an proangiogenic manner [45, 46]. Membrane-bound PR3 has not been detected in cultured mature endothelial cells so far. To our knowledge, this is the first investigation that reveals experimental evidence for mPR3 expression in Flk-1^+^ blood-derived myelomonocytic cells. We found antibody binding to mPR3 at higher percentages in GPA patients as compared to healthy controls, MPA, and SLE patients, respectively. In addition, immunoblot analysis of eEOC plasma membrane preparations confirmed mPR3 to be expressed by the cells. In contrast, MPA patients did not display increased percentages of mPR3^+^ cells and MPO expression did also not differ between the four groups. It has nevertheless to be mentioned that increased PR3 expression may partly result from apoptosis of myelomonocytic cells in GPA since PR3 has been shown to be externalized during neutrohil apoptosis [47].

The possible mechanistical relevance of these findings is highly speculative. Membrane-bound PR3 (mPR3), expressed in neutrophils, is targeted by antineutrophil cytoplasmic antibodies [2]. This interaction is controversially discussed to be responsible for degranulation of neutrophils and thereby for microvascular alteration [48–50]. Interestingly, increased PR3 membrane expression in GPA is typically found in generalised disease states while localised GPA is not associated with mPR3 upregulation in neutrophils [44]. Besides mediating these actions, mPR3 has also been demonstrated to be involved in apoptotic events. Pendergraft and colleagues found mPR3 to induce endothelial cell apoptosis via a mechanism that involves direct cleavage of p21, a major cell cycle inhibitor [51]. Comparable data had been reported in an earlier study [52], in which apoptosis of (bovine) endothelial cells was induced by proteinase 3 and elastase.

The fact that eEOC regeneration is impaired in GPA (and MPA) patients, an observation also made by other investigators [14], combined with the apparently increased antibody binding to PR3 in cells with endothelial properties potentially indicates a pathophysiological role for proteinase 3 in mediating suppression of endothelial-type cells.

In summary, with this study we clearly showed (i) impairment of the eEOC system in GPA, characterized by suppression of endothelial cell colony-forming and by lower percentages of peripheral circulating myelomonocytic cells with endothelial properties. Endothelial impairment may partially result from decreased Angiopoietin-1 levels in the blood. (ii) Since mPR3 is detected with higher percentages in total as well as in Flk-1^+^ myelomonocytic cells, this antigen could potentially serve as target of cANCA-induced endothelial cell dysfunction in GPA.

## Figures and Tables

**Table 1:** Patients' characteristics. A BVAS of 0 with a prednisolone dose below or equal to 7.5 mg/d defined remission while an active disease was defined by a BVAS ≥1. Patients with a BVAS of ≥8 were defined as highly active, a BVAS of <8 defined low activity. Diagnosis of GPA was according to the ACR criteria. Column 4 from the left displays the criteria that were fullfilled by an individual patient.

No.	Age	Diagnosis	ACR Criteria+	Previous drugs	PR3 (U/mL)	MPO (U/mL)	Creatinine (mg/dL)	CRP (mg/dL)	Maintenance therapy	BVAS	VDI	Gender	Disease duration	Renal histology	ANCA
1	75	GPA	Renal, lungs	Cyc	24	neg	3.54	18.1	Cyc, Prednisone	12	10	m	2 month	Renal involvement but no renal biopsy performed	1 : 80
2	66	GPA	ENT, lungs	Cyc, MTX	11	neg	0.93	10.5	Cyc, Prednisone	7	5	m	10 years	No biopsy	1 : 20
3	61	GPA	ENT, lungs, renal	Cyc, MTX	neg	neg	0.99	3.1	Prednisone	10	6	m	15 years	Focal segmental necrotizing glomerulonephritis	1 : 20
4	75	GPA	Lungs, renal	none	301	neg	3.31	112	Prednisone, PS	19	3	m	1 day	Focal segmental necrotizing glomerulonephritis	1 : 640
5	51	GPA	ENT, lungs	Cyc, MMF	neg	neg	0.9	3	Prednisone	5	5	f	4 years	Renal involvement but no renal biopsy performed	1 : 20
6	67	GPA	ENT, lungs, renal	Cyc, MTX	9	neg	1.2	5.7	Lefl, Prednisone	9	9	m	22 years	Renal involvement but no renal biopsy performed	1 : 40
7	56	GPA	ENT, lungs	Prednisone, AZA	25	neg	0.8	2	Prednisone, AZA	8	3	f	2 month	No biopsy	1 : 160
8	58	GPA	ENT, lungs	MTX	neg	neg	0.57	2	Prednisone, AZA	5	12	f	8 years	No biopsy	neg
9	62	GPA	ENT, lungs, renal	Cyc	neg	neg	1.02	2	Prednisone, AZA	4	5	m	8 years	Focal segmental necrotizing glomerulonephritis	neg
10	42	GPA	ENT, ENT granulomas	Cyc, MTX	11	neg	0.84	3.5	Cyc, prednisone	7	7	f	2 years	No biopsy	1 : 20
11	32	GPA	ENT, ENT granulomas	Cyc, AZA, Infli	5.3	neg	0.83	18.4	Prednisone, MMF	7	8	f	5 years	No biopsy	1 : 20
12	57	GPA	Lungs, renal	Cyc, MTX	neg	neg	1.72	3.3	Prednisone, MMF	10	7	m	12 years	Focal segmental necrotizing glomerulonephritis	1 : 20
13	30	GPA	Lungs, renal	Cyc, AZA, PP	6.4	neg	0.9	2.2	AZA	6	1	m	4 years	Focal segmental necrotizing extracapillary proliferating glomerulonephritis with intermediate interstitial nephritis	1 : 20
14	70	GPA	Lungs, renal	Cyc, AZA	neg	neg	4.14	2	Prednisone, AZA	10	8	f	8 years	Focal segmental necrotizing, extracapillary proliferating glomerulonephritis	neg
15	72	GPA	ENT, lungs	Cyc	88	neg	2.35	4.7	Prednisone, AZA	21	6	m	10 years	Renal involvement but no renal biopsy performed	1 : 320
16	25	GPA	ENT, renal	Cyc, PS	25	neg	1.14	4.5	Cyc, Prednisone	13	3	m	5 years	Focal segmental necrotizing, intra- and extracapillary proliferating glomerulonephritis	1 : 80
17	69	GPA	ENT, lungs	Cyc, AZA, MTX	6.8	neg	1.17	2	Prednisone	2	7	m	9 years	No biopsy	1 : 20
18	56	GPA	ENT, lungs, renal	Cyc, AZA	neg	neg	0.67	2	AZA	7	3	m	6 years	Focal segmental necrotizing glomerulonephritis	1 : 80
19	53	GPA	Lungs, renal	Cyc,AZA	5	neg	2.11	2.1	Prednisone, AZA	4	6	m	7 years	Focal segmental necrotizing extracapillary proliferating glomerulonephritis with mild interstitial nephritis	neg
20	84	GPA	ENT, lungs, renal	Cyc, Prednisone	12	neg	1.64	68	Prednisone	7	5	m	10 years	Renal involvement but no renal biopsy performed	1 : 40
21	83	GPA	Lungs, renal	MMF, Prednisone	7	neg	1.05	7	MMF, Prednisone	2	8	m	2 years	Renal involvement but no renal biopsy performed	1 : 20
22	80	GPA	Renal, renal histology	Cyc, Prednisone	5.5	neg	2.0	9.9	None	3	2	f	10 years	Focal segmental necrotizing. extracapillary proliferating glomerulonephritis	1 : 80
23	69	GPA	ENT, lungs, renal	Cyc, AZA	260	neg	1.08	6.4	AZA	4	4	f	6 years	Focal segmental glomerulosclerosis with chronic interstitial nephritis	1 : 320
24	65	GPA	ENT, lungs	Cyc, Prednisone	1.1	neg	0.83	7.1	Prednisone, MTX	3	5	f	5 years	No biopsy	neg
25	64	GPA	Lungs, renal	Cyc, PP, AZA	16	neg	1.94	5	Prednisone, MMF	9	9	f	4 month	Focal segmental necrotizing glomerulonephritis	1 : 40
26	71	MPA	Lungs, polyneuropathy, arthralgia	Cyc	neg	87	1.04	15.5	Prednisone	15	5	m	6 month	No biopsy	1 : 80
27	86	MPA	Lungs, renal, polyneuropathy	Cyc, PP	neg	134	0,79	40	Cyc	7	2	m	7 years	Focal segmental necrotizing glomerulonephritis	1 : 40
28	76	MPA	Renal	Cyc	neg	88	1.58	2	Prednisone	4	5	f	11 years	No biopsy	1 : 80
29		MPA	Lungs, poyneuropathy	Cyc, PS	neg	67	0.88	9.6	Prednisone, Cyc	15	3	m	3 month	No biopsy	1 : 40
30	76	MPA	Renal	MMF	neg	75	1.93	4.3	Prednisone, MMF	7	2	m	2 years	No biopsy	1 : 20
31	57	MPA	Arthralgia	Prednisone	neg	71	0.84	2	Prednisone	1	0	f	1 year	No biopsy	1 : 20
32	61	MPA	ESRD, lungs	AZA, MMF	neg	44	6.75	11.4	Prednisone	9	6	f	6 years	No biopsy	1 : 160
33	73	MPA	Lungs, renal, neurologic	Cyc, PP	neg	195	0.87	32.5	Prednisone, Cyc	13	10	f	1 year	No biopsy	1 : 320
34	72	MPA	Arthralgia	Prednisone	neg	110	1.02	2	none	2	2	f	4 years	No biopsy	1 : 160
35	86	MPA	Arthralgia	Cyc	neg	200	5.1	37.2	Prednisone, Cyc	14	4	f	8 years	No biopsy	1 : 160
